# Factors affecting disease progression in early-stage chronic kidney disease in a multi-ethnic, southeast Asian primary care population

**DOI:** 10.3389/fmed.2025.1526596

**Published:** 2025-08-19

**Authors:** Hui Qi Koh, Xueling Sim, Sky Wei Chee Koh

**Affiliations:** ^1^National University Polyclinics, National University Health System, Singapore, Singapore; ^2^Saw Swee Hock School of Public Health, National University of Singapore and National University Health System, Singapore, Singapore

**Keywords:** chronic kidney disease, CKD progression, primary care, risk factors, patient education

## Abstract

**Background:**

While effective risk factor control and medication optimization in early-stage CKD can significantly slow disease progression, a paucity of studies hinders comprehensive understanding. This study aims to identify factors associated with progression of early-stage CKD in primary care.

**Methods:**

We retrospectively analyzed data of CKD G1-G2 patients with type 2 diabetes or hypertension, recruited from an ongoing cohort between 2017 and 2023 from six polyclinics in Singapore. The outcome of interest was CKD progression, defined as a 25% decline in eGFR from baseline and worsening of CKD stage. Multivariable logistic regression was used to analyze the factors associated with CKD progression among early-stage CKD patients.

**Results:**

Among 19,274 patients analyzed, CKD progression occurred in 1,992 patients (10.3%). Patients had a mean age of 62.27 years (SD 9.54), 54.4% were male, 70.4% Chinese, 18.1% Malay, 8.2% Indian, and 3.4% Others. On multivariable analysis, factors associated with CKD progression include Malay ethnicity (OR: 1.52, 95% CI: 1.35, 1.72), A2 (OR: 1.41, 95% CI: 1.18, 1.70) and A3 albuminuria (OR: 4.19, 95% CI: 3.45, 5.10), diabetes (OR: 2.59, 95% CI: 2.18, 3.09), hypertension (OR: 1.69, 95% CI: 1.18, 2.41), increasing systolic BP (OR: 1.005, 95% CI 1.001, 1.008), active smoking (OR: 1.26, 95% CI: 1.09, 1.47), being on maximum doses of ACE inhibitors/ARBs at baseline (OR: 1.28, 95% CI 1.07, 1.53) and having undergone CKD counseling (OR: 1.84, 95% CI 1.59, 2.12). Increasing age (OR: 0.991, 95% CI 0.984, 0.998), higher baseline eGFR (OR: 0.968, 95% CI 0.965, 0.972), higher diastolic BP (OR: 0.989, 95% CI 0.983, 0.995), and BMI (OR: 0.981, 95% CI 0.971, 0.991) significantly reduced odds of CKD progression.

**Conclusion:**

This study identified key factors associated with early-stage CKD progression in a multi-ethnic Asian population. Further research is also needed to address benefits of patient counseling and SGLT2i use. Refining risk stratification methods will enable targeted interventions and improve outcomes for high-risk CKD patients.

## 1 Introduction

Chronic kidney disease (CKD) is characterized by a gradual loss of kidney function over time (1). Based on the Kidney Disease Improving Global Outcomes (KDIGO) guideline, CKD is defined as having either an estimated glomerular filtration rate (eGFR) < 60 mL/min/1.73 m^2^ or markers of kidney damage, including albuminuria, for more than 3 months (2). CKD remains a significant public health concern worldwide. Globally, it is estimated that about 10% of the general population or 843.6 million individuals are affected by CKD (3). Furthermore, progressive CKD is associated with higher risk of adverse clinical outcomes which include cardiovascular diseases, progression to end stage kidney failure and mortality (4). The economic burden of CKD is substantial, with studies projecting significant increases in annual direct costs that rise exponentially with disease progression, and renal replacement therapy-associated costs accounting for nearly half of the total costs (5, 6).

In Singapore, the age standardized prevalence of CKD has increased from 7.3% in 2019–2020 to 11.4% in 2021–2022 (7). Singapore ranks second in the world for diabetes induced kidney failure, second for prevalence and fifth for incidence of kidney failure (8). According to the Singapore renal registry, age standardized prevalence of definitive dialysis has increased from 949.0 to 1161.8 per million population between 2012 and 2022 (9). Among those on dialysis, diabetic nephropathy was the primary cause for end stage kidney failure (9).

Diabetes and hypertension remain the leading causes of CKD in many developed and developing countries (10). In a meta-analysis of 100 global studies, significant associations between diabetes and hypertension prevalence with CKD prevalence were reported (11). In Singapore, the prevalence of CKD was estimated to be 53% among adults with type 2 diabetes (12). A local projection study using the Markov model predicted an increase in CKD among residents, from 316,521 to 887,870, with a corresponding doubling of prevalence from 12.2 to 24.3% (13). This is further exacerbated by the high burden of chronic diseases and multimorbidity, which contributes to the escalating CKD prevalence.

Given the progressive and irreversible nature of CKD (14), slowing down disease progression remains the primary goal of most CKD treatment and management. This is often achieved through appropriate control of modifiable risk factors, dietary and lifestyle related, optimization of CKD medications such as angiotensin converting enzyme (ACE) inhibitors or angiotensin receptor blockers (ARBs) (15–17), and more recently sodium glucose cotransporter-2 (SGLT2) inhibitors (18, 19). The benefits and improvements in kidney outcomes associated with the use of ACE inhibitors/ARBs and SGLT2i have been well established and proven in many international studies (20–25). However, these were mainly conducted in populations with moderate to advanced CKD and studies to elucidate factors associated with progression among the early-stage CKD patients remain limited.

Primary care in Singapore, which is often patient’s first point of contact with the healthcare system, plays an important role in delaying CKD progression. As patients are usually asymptomatic in the early stages of CKD (26), active screening is vital in the early diagnosis and initiation of CKD treatment to facilitate preservation of kidney function and prevention of cardiovascular disease (26). To combat the growing CKD epidemic, the Singapore Ministry of Health (MOH) rolled out the Holistic Approach to Lowering and Tracking Kidney Disease (HALT-CKD) program in 2017 to all public primary care polyclinics with the aim of slowing down CKD progression to end stage kidney failure (27). The program uses a multi-pronged approach of prevention, education, treatment, and disease management to curb the rising prevalence of CKD (27). Primary objectives include (1) optimization of kidney protective medication such as ACE inhibitor or ARB, (2) ensuring control of diabetes and hypertension to target levels (28), and (3) CKD counseling by program coordinators. This program mainly included patients with type 2 diabetes or hypertension, as these conditions are the most common causes of early-stage CKD within primary care. Hence, this study aims to identify the factors associated with CKD progression among early-stage CKD patients with type 2 diabetes or hypertension in the primary care setting of Singapore to pinpoint high-risk groups and areas requiring targeted interventions, ultimately enhancing our population health strategy to better serve early-stage CKD patients in the region.

## 2 Materials and methods

### 2.1 Study design and study population

This was a retrospective study of patients recruited by six primary care polyclinics (National University Polyclinics (NUP)) in Singapore between 3 April 2017 and 31 March 2023 as part of the national HALT-CKD program. Patients recruited into the HALT-CKD program were aged 19 to 80 years old, with CKD stages G1 (eGFR ≥90 mL/min/1.73 m^2^) or G2 (eGFR 60-89 mL/min/1.73 m^2^) based on KDIGO staging criteria. Albuminuria stage (A1-3) was calculated based on KDIGO except stage A1, where we used gender specific cutoffs (Male: < 2.5 mg/mmol, Female: < 3.5 mg/mmol, respectively) (29).

### 2.2 Data and variables measurement

**Figure 1:**
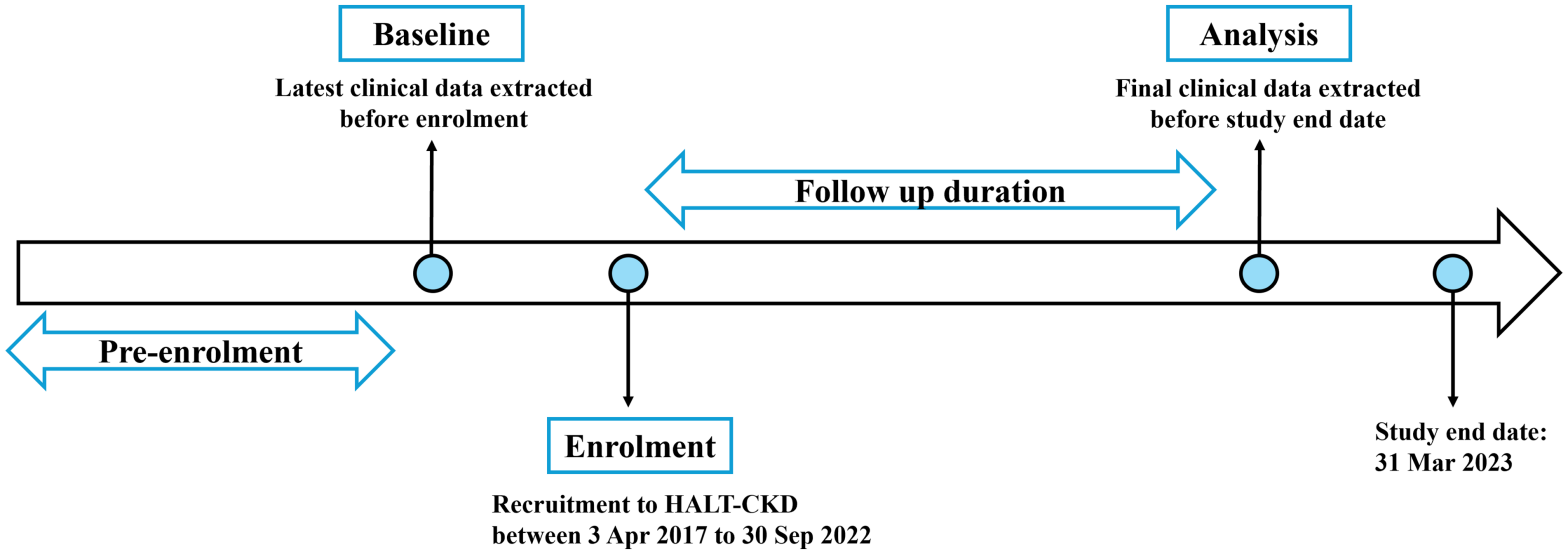
Outline of study timeline and data collection points.

The study dataset included patient information such as age, sex, diagnosis, medications, and dosages and laboratory measurements. Clinical data were captured at two time points: at baseline defined as the last measurement before enrollment into the program and at analysis defined as the final measurement extracted prior to study end date of 31 March 2023. Baseline eGFR of each patient was calculated using the mean of up to four pre-enrolment serum creatinine measurements using the Chronic Kidney Disease Epidemiology (CKD-EPI) 2009 equation (30). Variables related to the HALT-CKD program such as patient’s enrolment date, counseling status, and other program-specific metrics were tracked. Figure 1 summarizes the data collection time points and timeline of this study.

### 2.3 Study inclusion and exclusion

**Figure 2:**
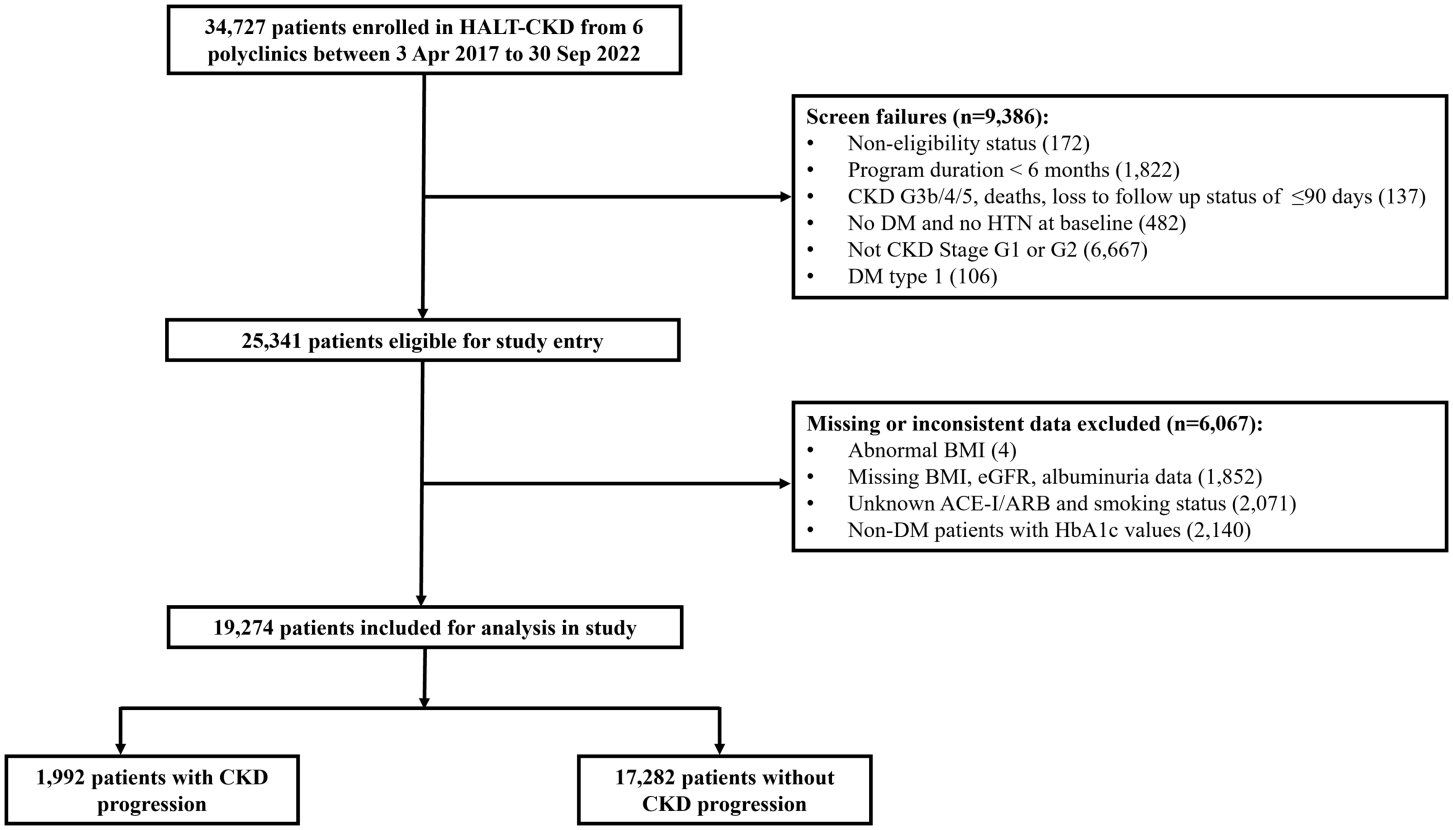
Flowchart of patients included in this study.

Within the HALT-CKD program, patients with the following criteria were included for analysis: (1) with existing diagnosis of hypertension or type 2 diabetes mellitus, (2) stage G1 or G2 based on KDIGO definition, with persistently elevated albuminuria ≥3 mg/mmol for more than 3 months, and (3) with a minimum program duration of 6 months. Figure 2 shows the flowchart of patients included in the study.

### 2.4 Outcome definition

The outcome of interest was CKD progression, defined by a 25% or more reduction in eGFR from baseline, and worsening of CKD stage based on KDIGO guideline (2). CKD progression was determined based on individual’s percentage change in eGFR from baseline to final measurement. Patients who had improved eGFR category, same eGFR category despite a 25% or more reduction in eGFR or less than 25% reduction in eGFR from baseline were defined as not having CKD progression.

### 2.5 Statistical analyses

All statistical analysis was performed using R 4.3.1. Statistical significance was assessed at *p* < 0.05 using a two-tailed test. Descriptive statistics were performed with categorical variables expressed as counts and percentages and continuous variables expressed as mean and standard deviation. Patient characteristics were analyzed using Pearson chi-square test for categorical variables and Student’s *t*-test for continuous variables. The dependent variable was CKD progression following KDIGO definition (2). Potential factors studied include age, sex, ethnicity, polyclinic, CKD counseling status, body mass index (BMI), albuminuria status, HbA1c, CKD medications such as ACE inhibitor/ARB and SGLT2i, systolic and diastolic blood pressure (SBP and DBP), albuminuria, presence of chronic diseases such as hypertension, diabetes, and hyperlipidemia, and smoking. Multivariable logistic regression was performed using CKD progression as the outcome of interest, adjusted for all potential explanatory variables except HbA1c, which was excluded to prevent singularity error due to its close association with another variable, presence of diabetes. To ensure the absence of multicollinearity, variance inflation factors (VIFs) and tolerance values were calculated for all predictor variables, confirming that none exceeded the threshold for concern (VIF < 5, tolerance > 0.2). The results were expressed as odds ratios (ORs) with their 95% confidence intervals (CI) for multivariable analysis after adjustments.

### 2.6 Ethics approval

Ethics approval was obtained from National Healthcare Group (NHG) Domain Specific Review Board (DSRB) on 29 June 2023, NHG DSRB Reference: 2023/00314. Waiver of consent was approved for the conduct of this study and all patient data were de-identified prior to study analysis.

## 3 Results

A total of 34,727 patients were enrolled in HALT-CKD from the six polyclinics between 3 Apr 2017 and 30 Sep 2022. After excluding 6,067 patients due to CKD stage, missing or inconsistent data, 19,274 early-stage CKD patients were analyzed with 1,992 (10.3%) having CKD progression. Table 1 shows the patient characteristics by CKD progression status.

**Table 1:** Characteristics of patients by CKD progression status.

	Overall	CKD progression	No CKD progression	*p*-value
	(*n* = 19,274)	(*n* = 1,992)	(*n* = 17,282)	
Mean age (years) (SD)	62.27 (±9.54)	63.50 (±8.85)	62.13 (±9.60)	<0.001^a^
Gender, *n* (%)				0.60^b^
Male	10,489 (54.4)	1,095 (55.0)	9,394 (54.4)	
Female	8,785 (45.6)	897 (45.0)	7,888 (45.6)	
Ethnicity, *n* (%)				<0.001^b^
Chinese	13,565 (70.4)	1,299 (65.2)	12,266 (71.0)	
Malay	3,482 (18.1)	492 (24.7)	2,990 (17.3)	
Indian	1,578 (8.2)	141 (7.1)	1,437 (8.3)	
Others	649 (3.4)	60 (3.0)	589 (3.4)	
Polyclinic, *n* (%)				<0.001^b^
Polyclinic A	4,497 (23.3)	522 (26.2)	3,975 (23.0)	
Polyclinic B	3,430 (17.8)	373 (18.7)	3,057 (17.7)	
Polyclinic C	2,779 (14.4)	312 (15.7)	2,467 (14.3)	
Polyclinic D	3,814 (19.8)	339 (17.0)	3,475 (20.1)	
Polyclinic E	2,675 (13.9)	176 (8.8)	2,499 (14.5)	
Polyclinic F	2,079 (10.8)	270 (13.6)	1,809 (10.5)	
Smoking status, *n* (%)				0.011^b^
Non-smoker	14,626 (75.9)	1,461 (73.3)	13,165 (76.2)	
Smoker	2,604 (13.5)	309 (15.5)	2,295 (13.3)	
Ex-smoker	2,044 (10.6)	222 (11.1)	1,822 (10.5)	
Mean eGFR (mL/min/1.73 m^2^) (SD)	85.79 (±14.17)	80.36 (±13.18)	86.41 (±14.15)	<0.001^a^
Baseline CKD Stage, *n* (%)				<0.001^b^
Stage G1 (eGFR ≥90 mL/min/1.73 m^2^)	7,953 (41.3)	569 (28.6)	7,384 (42.7)	
Stage G2 (eGFR 60–89 mL/min/1.73 m^2^)	11,321 (58.7)	1,423 (71.4)	9,898 (57.3)	
Baseline albuminuria, *n* (%)				<0.001^b^
Normo (A1)	2,376 (12.3)	149 (7.5)	2,227 (12.9)	
Micro (A2)	14,116 (73.2)	1,174 (58.9)	12,942 (74.9)	
Macro (A3)	2,782 (14.4)	669 (33.6)	2,113 (12.2)	
Presence of diabetes at baseline, *n* (%)	15,532 (80.6)	1,820 (91.4)	13,712 (79.3)	<0.001^b^
Presence of hypertension at baseline, *n* (%)	18,552 (96.3)	1,956 (98.2)	16,596 (96.0)	<0.001^b^
Presence of hyperlipidemia at baseline, *n* (%)	18,499 (96.0)	1,955 (98.1)	16,544 (95.7)	<0.001^b^
Mean baseline HbA1c (%) (SD)	7.49 (±1.37)	7.71 (±1.57)	7.46 (±1.34)	<0.001^a^
Mean baseline systolic BP (mmHg) (SD)	133.15 (±14.53)	134.26 (±15.24)	133.02 (±14.44)	<0.001^a^
Mean baseline diastolic BP (mmHg) (SD)	73.51 (±9.24)	72.81 (±9.69)	73.59 (±9.19)	<0.001^a^
Mean BMI (kg/m^2^) (SD)	27.47 (±5.52)	27.19 (±5.37)	27.50 (±5.53)	0.009^a^
ACE inhibitor/ARB at baseline, *n* (%)				<0.001^b^
Not on ACE inhibitor/ARB	3,131 (16.2)	206 (10.3)	2,925 (16.9)	
Not on max dose	11,303 (58.7)	1,075 (54.0)	10,228 (59.2)	
Max dose	4,840 (25.1)	711 (35.7)	4,129 (23.9)	
SGLT2i use, *n* (%)	5,961 (30.9)	727 (36.5)	5,234 (30.3)	<0.001^b^
Counseled on CKD, *n* (%)	14,940 (77.5)	1,737 (87.2)	13,203 (76.4)	<0.001^b^

a Student’s *t*-test.

b Chi-square test.

BP, blood pressure; ACE inhibitor, angiotensin-converting enzyme inhibitor; ARB, angiotensin receptor blocker; SGLT2i, sodium glucose co-transport 2 inhibitor; BMI, body mass index.

Patients with CKD progression were older (mean difference 1.371 years, 95% CI 0.957, 1.785) and had a lower baseline eGFR (mean difference 6.05 mL/min/1.73 m^2^, 95% CI 5.43, 6.67) than those without progression (Table 1). The following variables were found to be significantly associated with CKD progression: ethnicity (*χ^2^* = 66.7, *p* < 0.001), polyclinic seen (*χ^2^* = 76.4, *p* < 0.001), smoking status (*χ^2^* = 8.64, *p* = 0.011), baseline CKD (*χ^2^* = 147.8, *p* < 0.001) and albuminuria stage (*χ^2^* = 668.9, *p* < 0.001), presence of diabetes (*χ^2^* = 165.0, *p* < 0.001), hypertension (*χ^2^* = 23.2, *p* < 0.001) and hyperlipidemia (*χ^2^* = 26.9, *p* < 0.001) at baseline, use of ACE inhibitors/ARBs at baseline (*χ^2^* = 155.0, *p* < 0.001), ever used SGLT2i (*χ^2^* = 32.2, *p* < 0.001), and ever been counseled for CKD (*χ^2^* = 119.6, *p* < 0.001). Patients with CKD progression were also found to have higher baseline HbA1c (mean difference 0.25, 95% CI 0.19, 0.32%), higher baseline systolic BP (mean difference 1.23 mmHg, 95% CI 0.53 mmHg, 1.94 mmHg), lower baseline diastolic BP (mean difference −0.79 mmHg, 95% CI −1.23 mmHg, −0.34 mmHg), and lower BMI (mean difference −0.13 kg/m^2^, 95% CI −0.56 kg/m^2^, −0.05 kg/m^2^). No significant associations between gender and CKD progression were found (*χ^2^* = 0.27, *p* = 0.60).

On multivariate analysis, the odds of CKD progression were reduced (OR: 0.991, 95% CI: 0.984, 0.998) with each year increase in age after adjustments (Table 2). Ethnicity was a significant factor for CKD progression, with Malays being 52% more likely to progress compared to Chinese (OR: 1.52, 95% CI: 1.35, 1.72). The odds of CKD progression differed across polyclinics (*p* < 0.001), with Polyclinic D (OR: 0.80, 95% CI: 0.68, 0.94) and E (OR: 0.58, 95% CI 0.48, 0.70) at lower odds of CKD progression, and Polyclinic F at higher odds of CKD progression (OR: 1.33, 95% CI 1.11, 1.59), compared to Polyclinic A. Smoking was a significant factor for CKD progression with current smokers having a higher odds of progression (OR: 1.26, 95% CI: 1.09, 1.47) than non-smokers.

**Table 2:** Multivariable regression analysis showing variables associated with CKD progression.

	*p*-value	OR	95% CI	
			Lower	Upper
Age	0.013	0.991	0.984	0.998
Male (ref = female)	0.058	1.12	1.00	1.25
Ethnicity	<0.001			
Chinese		1		
Malay	<0.001	1.52	1.35	1.72
Indian	0.538	0.94	0.78	1.14
Others	0.593	1.08	0.81	1.44
Polyclinic	<0.001			
Polyclinic A		1		
Polyclinic B	0.061	1.12	0.99	1.34
Polyclinic C	0.473	1.06	0.90	1.26
Polyclinic D	0.006	0.80	0.68	0.94
Polyclinic E	<0.001	0.58	0.48	0.70
Polyclinic F	0.002	1.33	1.11	1.59
Smoking status	0.009			
Non-smoker		1		
Smoker	0.002	1.26	1.09	1.47
Ex-smoker	0.704	1.03	0.87	1.22
Baseline eGFR (mL/min/1.73 m^2^)	<0.001	0.968	0.965	0.972
Baseline albuminuria	<0.001			
Normo (A1)		1		
Micro (A2)	<0.001	1.41	1.18	1.70
Macro (A3)	<0.001	4.19	3.45	5.10
Presence of diabetes at baseline (ref = no diabetes)	<0.001	2.59	2.18	3.09
Presence of hypertension at baseline (ref = no hypertension)	0.004	1.69	1.18	2.41
Presence of hyperlipidemia at baseline (ref = no hyperlipidemia)	0.327	1.19	0.84	1.70
Baseline systolic BP (mmHg)	0.017	1.005	1.001	1.008
Baseline diastolic BP (mmHg)	<0.001	0.989	0.983	0.995
BMI (kg/m^2^)	<0.001	0.981	0.971	0.991
ACE-I/ARB use at baseline	0.001			
Not on ACE-I/ARB		1		
Not on max dose	0.495	1.06	0.90	1.25
Max dose	0.006	1.28	1.07	1.53
SGLT2i (ref = not on SGLT2i)	0.601	1.03	0.92	1.15
Counseling status (ref = not counseled)	<0.001	1.84	1.59	2.12

BP, blood pressure; ACE inhibitor, angiotensin-converting enzyme inhibitor; ARB, angiotensin receptor blocker; SGLT2i, sodium glucose co-transport 2 inhibitor; BMI, body mass index.

Higher baseline eGFR was associated with lower odds of CKD progression (OR: 0.968, 95% CI: 0.965, 0.972) (Table 2). We observed a stepwise increase in CKD progression risk with albuminuria severity: patients with Stage A3 albuminuria (OR: 4.19, 95% CI: 3.45, 5.10) and Stage A2 albuminuria (OR: 1.41, 95% CI: 1.18, 1.70) had higher odds of CKD progression, compared to patients with Stage A1 albuminuria (Table 2). The presence of chronic conditions such as diabetes (OR: 2.59, 95% CI: 2.18, 3.09) or hypertension (OR: 1.69, 95% CI: 1.18, 2.41) at baseline significantly increased the odds of CKD progression (Table 2). While increasing baseline systolic BP was found to significantly increase odds of CKD progression (OR: 1.005, 95% CI 1.001, 1.008), increasing baseline diastolic BP was found to significantly reduce odds of CKD progression (OR: 0.989, 95% CI 0.983, 0.995). For BMI, the odds of CKD progression were lower (OR: 0.981, 95% CI: 0.971, 0.991) with each unit increase in BMI.

In terms of medications, patients who were on maximal doses of ACE inhibitor/ARB demonstrated significantly increased odds of CKD progression compared to those who were not on ACE inhibitor/ARB (OR: 1.28, 95% CI 1.07, 1.53) (Table 2). SGLT2i use did not show statistically significant association with CKD progression following adjustments (*p* = 0.601). CKD counseling remained significantly associated with increased odds of CKD progression (OR: 1.84, 95% CI: 1.59, 2.12).

## 4 Discussion

CKD progression occurred in 10.3% of the early-stage CKD patients analyzed in this study. On multivariable analysis, factors associated with increased odds of progression included Malay ethnicity, worsening albuminuria stage, presence of chronic diseases such as diabetes and hypertension, higher baseline systolic BP, smoking status, and baseline use of ACE inhibitor/ARB at maximal doses. Increasing age, higher baseline eGFR, diastolic BP, and BMI showed reduced odds of progression among CKD G1-G2 patients. Additionally, polyclinic location was significantly associated with CKD progression. This may be attributed to inherent differences in sociodemographic distribution and practice patterns across locations that were not fully accounted for in this study.

In general, findings from this study align well with established risk factors for CKD progression. A systematic review and meta-analysis identified male sex, proteinuria and diabetes, as risk factors for the development and progression of CKD (31). Proteinuria, smoking, hypertension, and low HDL cholesterol are associated with accelerated disease progression in early CKD (32). Gender differences in risk factors have been observed, with proteinuria being the most crucial factor for males and poor glycemic control for females (33). Poor blood pressure control is a shared risk factor for both genders (33). Presently, systematic reviews on CKD were mainly conducted on CKD cohorts with moderate to advanced CKD. Similarly, risk prediction tools for CKD such as the Kidney Failure Risk Equation, which predicts an individual’s risk for end stage kidney disease is only validated for use in those with CKD stages G3–G5 (34, 35) and not the earlier stages. Although there are promising attempts to develop risk prediction models for early-stage CKD (36) and its progression, focus was primarily on the diabetic population (37–39) and may not be used exclusively for early-stage CKD (39). With the evolving shift in care model toward preventive health at the population level, further research including systematic review studies focusing on early-stage CKD could be performed to better inform upstream management of the disease.

International studies on early-stage CKD cohorts have reported ethnic disparities in outcomes, with ethnic minorities being the most adversely affected (40, 41). A unique characteristic of Singapore is its multiethnic makeup comprising predominantly the Chinese, Indian, Malays and other communities. This study results revealed that ethnicity was a significant factor for early-stage CKD progression with Malays reportedly being most at risk. This was consistent with results from existing local CKD studies where Malays were shown to have higher odds of CKD (42), more rapid progression to end stage renal disease (43), and disproportionately higher incident CKD compared to other ethnic groups (44). In our study, Malays had a higher proportion of ever smokers (28.3%), current and ex-smokers combined, compared to Chinese (23.0%), Indian (22.9%), and others (26.8%). There was also a higher proportion of patients with Stage A3 albuminuria status at baseline among the Malays (18.2%) compared to other ethnic groups (Chinese: 13.6%, Indian: 12.4%, Others: 16.8%). Furthermore, the mean BMI of Malays was found to be highest (30.26 kg/m^2^) among all ethnic groups (Chinese: 26.40 kg/m^2^, Indian: 27.90 kg/m^2^, Others: 29.19 kg/m^2^). Studies have established obesity as a risk factor for the onset and progression of CKD (45, 46). Obesity can exacerbate CKD through pathophysiological processes of inflammation, oxidative stress, endothelial dysfunction, and proteinuria (46). These disparities may be influenced by differences in health beliefs, socioeconomic status, and health system engagement, highlighting the need for further research and comprehensive interventions to address these disparities.

Although the kidney protective effects of ACE inhibitors/ARBs have been well established in many CKD studies, these were mostly conducted on mid to advanced staged CKD patients (21, 47). One study that investigated the effects of ACE inhibitors/ARBs showed significantly reduced CKD progression among those who were on ACE inhibitors/ARBs, even among early-stage patients (20). While studies have demonstrated a dose–response relationship for certain ACE inhibitors/ARBs such as irbesartan (15), benazepril and losartan (48) with better kidney outcomes reported at higher dosages, the same effect was not seen in this study, which revealed that early-stage CKD patients had increased odds of progression at maximal dosages of ACE inhibitor/ARB at baseline. A possible reason for this observed discrepancy may be the differing clinical characteristics of patients who were on maximum ACE inhibitor/ARB dose compared to those who were not. Patients who were on maximum ACE inhibitor/ARB dose had persistently higher mean SBP: 135.0 mmHg (Not on max dose: 133.1 mmHg, Not on ACE inhibitor/ARB: 130.4 mmHg) and a higher proportion (25.3%) with Stage A3 albuminuria levels compared to the rest at baseline (Not on max dose: 12.6%, Not on ACE inhibitor/ARB: 4.2%). Furthermore, the proportion of patients with concomitant SGLT2i use was highest among the group on maximum ACE inhibitor/ARB doses (42.3%) (Not on max dose: 28.6%, Not on ACE inhibitor/ARB: 21.7%). We speculate that patients on maximum doses may have had more complex or advanced disease, with inadequate response to therapy despite maximal dosing, attributed to various factors such as medication non-adherence, need for additional therapies to control albuminuria and blood pressure, or underlying disease severity and etiology. Further investigation is needed to understand the relationship between ACE inhibitor/ARB dosing and CKD progression in this population.

Contrary to study’s expectation, the provision of CKD counseling by program coordinators did not lower the odds of progression in early-stage CKD patients. Counseling was instead associated with increased odds of progression. Admittedly, challenges in patient education efforts exist. While education may improve the knowledge and literacy of positive health practices in patients, this may not necessarily translate to optimal adoption of health behaviors leading to better health outcomes. This was demonstrated in findings from a systematic review where a consistent relationship between health literacy and positive self-management behaviors in CKD patients was shown but association between health literacy and health outcomes remained inconclusive and poorly understood (49). A further limitation that may have impeded the effectiveness of counseling was that program coordinators only delivered the education once or twice to patients in their entire program duration. Research suggests that providing risk information at a single time point may be insufficient (50), highlighting the need for repeated or ongoing educational interventions to reinforce patient understanding and promote behavior change.

This study has several strengths, including a robust sample size that provides ample power to detect significant effects. The large, multi-ethnic patient cohort offers a representative real-world snapshot of early-stage CKD patients in the southeast Asian region, enhancing the generalizability of the findings to this diverse population. The analysis was based on a comprehensive dataset combining clinical data from medical records and detailed documentation from program coordinators. This approach minimized missing data and ensured robust follow-up, facilitated by the program’s mandatory national reporting requirements, which ensured a high level of data completeness and accuracy. There are limitations to be acknowledged in this study. Firstly, as an observational study, we could not control the time between baseline and outcome determination, resulting in varying lengths of time between baseline and final measurement. This variability could impact results and introduce significant variation. To mitigate potential bias, we enforced a minimum 6-month observation period, ensuring a more accurate representation of disease progression and program response. Secondly, missing data, primarily due to incorrect BMI recordings from machine errors, were excluded from analysis. Despite manual imputation of height values, some BMI values remained unresolved, particularly for patients with mobility issues. To maintain data integrity, patients with incomplete records were excluded. Multiple imputation methods were not employed due to concerns of introducing biases into regression analysis, particularly regarding BMI accuracy. Thirdly, this study would have missed patients who were on follow up with private general practitioner clinics or nephrologists for their CKD. Fourthly, some extent of selection bias may be introduced due to exclusion of patients with pre-diabetes (due to predefined inclusion criteria for the original cohort) and missing clinical parameters. Lastly, some variables that were important for CKD progression were not collected by the program and hence could not be studied. These included information on patient’s diet, physical activity, medication adherence, LDL-Cholesterol levels, statin medication use, socioeconomic status, other cardiovascular co-morbidities, aetiology and family history of CKD. The study would have been strengthened by collecting data on these four variables at baseline (SGLT2i use, CKD counseling, BMI, smoking status), allowing for more accurate assessment of their impact on CKD progression.

## 5 Conclusion

Our study explored the factors affecting CKD progression among the early-stage CKD patients in a multi-ethnic population. Findings from this study mostly concur with existing evidence. Early-stage CKD patients who present with diabetes, hypertension, substantial proteinuria, are current smokers, of Malay ethnicity, and required maximum doses of ACE inhibitor/ARB at baseline were shown to have increased odds of CKD progression. This further supports the continued use of ACE inhibitor/ARB as a first line therapy in CKD management, including those of early-stage CKD. The study has also highlighted potential gaps in existing CKD counseling practices and usage of SGLT2i which may benefit from future evaluations to better assess its constraints. With appropriate interventions and management of risk factors, disease progression can be delayed in the early stages of CKD.

## Data Availability

The raw data supporting the conclusions of this article will be made available by the authors, without undue reservation.

## References

[1] Levey AS Coresh J Balk E Kausz AT Levin A Steffes MW. National kidney foundation practice guidelines for chronic kidney disease: evaluation, classification, and stratification. Ann Intern Med. (2003) 139:137–47. doi: 10.7326/0003-4819-139-2-200307150-00013 12859163

[2] Stevens PE Ahmed SB Carrero JJ Foster B Francis A Hall RK. KDIGO 2024 clinical practice guideline for the evaluation and management of chronic kidney disease. Kidney Int. (2024) 105:S117–314. doi: 10.1016/j.kint.2023.10.018 38490803

[3] Kovesdy CP. Epidemiology of chronic kidney disease: an update 2022. Kidney Int Suppl. (2022) 12:7–11. doi: 10.1016/j.kisu.2021.11.003, PMID: 35529086 PMC9073222

[4] Sud M Tangri N Pintilie M Levey AS Naimark D. Risk of end-stage renal disease and death after cardiovascular events in chronic kidney disease. Circulation. (2014) 130:458–65. doi: 10.1161/CIRCULATIONAHA.113.007106, PMID: 24899688

[5] Chadban S Arıcı M Power A Wu MS Mennini FS Arango Álvarez JJ. Projecting the economic burden of chronic kidney disease at the patient level (inside CKD): a microsimulation modelling study. eClinicalMedicine. (2024) 72:102615. doi: 10.1016/j.eclinm.2024.102615, PMID: 39010976 PMC11247148

[6] Jha V Al-Ghamdi SM Li G Wu MS Stafylas P Retat L. Global economic burden associated with chronic kidney disease: a pragmatic review of medical costs for the inside CKD research programme. Adv Ther. (2023) 40:4405–20. doi: 10.1007/s12325-023-02608-9, PMID: 37493856 PMC10499937

[7] Epidemiology & Disease Control Division and Policy. (2023). Available online at: https://www.moh.gov.sg/docs/librariesprovider5/resources-statistics/reports/nphs-2022-survey-report-(final).pdf (Accessed April 21, 2024)

[8] United States Renal Data System. USRDS Annual Data Report: epidemiology of kidney disease in the United States. US Department of Health and Human Services. Available online at: https://usrds-adr.niddk.nih.gov/2023/end-stage-renal-disease/11-international-comparisons (Accessed April 21, 2024)

[9] National Registry of Diseases Office, Health Promotion Board, Singapore. Singapore renal registry annual report 2022. (2024). Available online at: https://www.nrdo.gov.sg/docs/librariesprovider3/default-document-library/srr-annual-report-2022.pdf?sfvrsn=5ca01be2_0 (Accessed April 21, 2024)

[10] Jha V Garcia-Garcia G Iseki K Li Z Naicker S Plattner B. Chronic kidney disease: global dimension and perspectives. Lancet. (2013) 382:260–72. doi: 10.1016/S0140-6736(13)60687-X, PMID: 23727169

[11] Hill NR Fatoba ST Oke JL Hirst JA O’Callaghan CA Lasserson DS. Global prevalence of chronic kidney disease–a systematic review and meta-analysis. PLoS One. (2016) 11:e0158765. doi: 10.1371/journal.pone.0158765 27383068 PMC4934905

[12] Low SK Sum CF Yeoh LY Tavintharan S Ng XW Lee SB. Prevalence of chronic kidney disease in adults with type 2 diabetes mellitus. Ann Acad Med Singap. (2015) 44:164–71. doi: 10.47102/annals-acadmedsg.V44N5p164 26198322

[13] Wong LY Liew AS Weng WT Lim CK Vathsala A Toh MP. Projecting the burden of chronic kidney disease in a developed country and its implications on public health. Int J Nephrol. (2018) 2018:5196285. doi: 10.1155/2018/5196285 30112209 PMC6077589

[14] López-Novoa JM Martínez-Salgado C Rodríguez-Peña AB Hernández FJ. Common pathophysiological mechanisms of chronic kidney disease: therapeutic perspectives. Pharmacol Ther. (2010) 128:61–81. doi: 10.1016/j.pharmthera.2010.05.006 20600306

[15] Parving HH Lehnert H Bröchner-Mortensen J Gomis R Andersen S Arner P. The effect of irbesartan on the development of diabetic nephropathy in patients with type 2 diabetes. N Engl J Med. (2001) 345:870–8. doi: 10.1056/NEJMoa011489, PMID: 11565519

[16] Iino Y Hayashi M Kawamura T Shiigai T Tomino Y Yamada K. Renoprotective effect of losartan in comparison to amlodipine in patients with chronic kidney disease and hypertension—a report of the Japanese losartan therapy intended for the global renal protection in hypertensive patients (JLIGHT) study. Hypertens Res. (2004) 27:21–30. doi: 10.1291/hypres.27.21 15055252

[17] Maschio G Alberti D Locatelli F Mann JF Motolese M Ponticelli C. Angiotensin-converting enzyme inhibitors and kidney protection: the AIPRI trial. J Cardiovasc Pharmacol. (1999) 33:S16–20. doi: 10.1097/00005344-199900001-00004 10028949

[18] Heerspink HJ Karasik A Thuresson M Melzer-Cohen C Chodick G Khunti K. Kidney outcomes associated with use of SGLT2 inhibitors in real-world clinical practice (CVD-REAL 3): a multinational observationalcohort study. Lancet Diabetes Endocrinol. (2020) 8:27–35. doi: 10.1016/S2213-8587(19)30384-5 31862149

[19] EMPA-Kidney Collaborative Group Herrington WG Staplin N Wanner C Green JB. Empagliflozin in patients with chronic kidney disease. N Engl J Med. (2023) 388:117–27. doi: 10.1056/NEJMoa2204233, 36331190 PMC7614055

[20] Zheng CM Wang JY Chen TT Wu YC Wu YL Lin HT. Angiotensin-converting enzyme inhibitors or angiotensin receptor blocker monotherapy retard deterioration of renal function in Taiwanese chronic kidney disease population. Sci Rep. (2019) 9:2694. doi: 10.1038/s41598-019-38991-z 30804406 PMC6389886

[21] Jafar TH Stark PC Schmid CH Landa M Maschio G De Jong PE. Progression of chronic kidney disease: the role of blood pressure control, proteinuria, and angiotensin-converting enzyme inhibition: a patient-level meta-analysis. Ann Intern Med. (2003) 139:244–52. doi: 10.7326/0003-4819-139-4-200308190-00006, PMID: 12965979

[22] Neuen BL Young T Heerspink HJ Neal B Perkovic V Billot L. SGLT2 inhibitors for the prevention of kidney failure in patients with type 2 diabetes: a systematic review and meta-analysis. Lancet Diabetes Endocrinol. (2019) 7:845–54. doi: 10.1136/bmj.m4573 31495651

[23] Fernandez-Fernandez B Sarafidis P Kanbay M Navarro-Gonzalez JF Soler MJ Górriz JL. SGLT2 inhibitors for non-diabetic kidney disease: drugs to treat CKD that also improve glycaemia. Clin Kidney J. (2020):728–33. doi: 10.1093/ckj/sfaa198, PMID: 33123352 PMC7577767

[24] Lewis EJ Hunsicker LG Clarke WR Berl T Pohl MA Lewis JB. Renoprotective effect of the angiotensin-receptor antagonist irbesartan in patients with nephropathy due to type 2 diabetes. N Engl J Med. (2001) 345:851–60. doi: 10.1056/NEJMoa011303, PMID: 11565517

[25] Liu AY Low S Yeoh E Lim EK Renaud CJ Teoh ST. A real-world study on SGLT2 inhibitors and diabetic kidney disease progression. Clin Kidney J. (2022) 15:1403–14. doi: 10.1093/ckj/sfac044, PMID: 35756732 PMC9217649

[26] Ramakrishnan C Tan NC Yoon S Hwang SJ Foo MW Paulpandi M. Healthcare professionals’ perspectives on facilitators of and barriers to CKD management in primary care: a qualitative study in Singapore clinics. BMC Health Serv Res. (2022) 22:560. doi: 10.1186/s12913-022-07949-9 35473928 PMC9044787

[27] MOH News highlights. Available online at: https://www.moh.gov.sg/news-highlights/details/speech-by-mr-chan-heng-kee-permanent-secretary-ministry-of-health-at-the-3rd-kidney-care-conference-2019-25-may-2019 (Accessed April 21, 2024)

[28] Ooi XY Yeo SC. How can we HALT-CKD in primary care? Clinical care paths to manage chronic kidney disease. Singapore Fam Physician. (2021) 47:13–8. doi: 10.33591/sfp.47.8.u3

[29] Lambers Heerspink HJ Brantsma AH de Zeeuw D Bakker SJ de Jong PE Gansevoort RT. Albuminuria assessed from first-morning-void urine samples versus 24-hour urine collections as a predictor of cardiovascular morbidity and mortality. Am J Epidemiol. (2008) 168:897–905. doi: 10.1093/aje/kwn209 18775924

[30] Levey AS Stevens LA Schmid CH Zhang Y Castro AF III Feldman HI. A new equation to estimate glomerular filtration rate. Ann Intern Med. (2009) 150:604–12. doi: 10.7326/0003-4819-150-9-200905050-00006 19414839 PMC2763564

[31] Tsai WC Wu HY Peng YS Ko MJ Wu MS Hung KY. Risk factors for development and progression of chronic kidney disease: a systematic review and exploratory meta-analysis. Medicine. (2016) 95:e3013. doi: 10.1097/MD.0000000000003013, PMID: 26986114 PMC4839895

[32] Yoshida T Takei T Shirota S Tsukada M Sugiura H Itabashi M. Risk factors for progression in patients with early-stage chronic kidney disease in the Japanese population. Intern Med. (2008) 47:1859–64. doi: 10.2169/internalmedicine.47.1171, PMID: 18981628

[33] Chang PY Chien LN Lin YF Wu MS Chiu WT Chiou HY. Risk factors of gender for renal progression in patients with early chronic kidney disease. Medicine. (2016) 95:e4203. doi: 10.1097/MD.0000000000004203, PMID: 27472690 PMC5265827

[34] Wang Y Nguyen FN Allen JC Lew JQ Tan NC Jafar TH. Validation of the kidney failure risk equation for end-stage kidney disease in Southeast Asia. BMC Nephrol. (2019) 20:451. doi: 10.1186/s12882-019-1643-0 31801468 PMC6894117

[35] Tangri N Stevens LA Griffith J Tighiouart H Djurdjev O Naimark D. A predictive model for progression of chronic kidney disease to kidney failure. JAMA. (2011) 305:1553–9. doi: 10.1001/jama.2011.45 21482743

[36] Shih CC Lu CJ Chen GD Chang CC. Risk prediction for early chronic kidney disease: results from an adult health examination program of 19,270 individuals. Int J Environ Res Public Health. (2020) 17:4973. doi: 10.3390/ijerph17144973, PMID: 32664271 PMC7399976

[37] Low S Lim SC Zhang X Zhou S Yeoh LY Liu YL. Development and validation of a predictive model for chronic kidney disease progression in type 2 diabetes mellitus based on a 13-year study in Singapore. Diabetes Res Clin Pract. (2017) 123:49–54. doi: 10.1016/j.diabres.2016.11.008 27923172

[38] Fu Z Wang Z Clemente K Jaisinghani M Poon KM Yeo AW. Development and deployment of a nationwide predictive model for chronic kidney disease progression in diabetic patients. Front Nephrol. (2024) 3:1237804. doi: 10.3389/fneph.2023.1237804, PMID: 38260055 PMC10800693

[39] Surian NU Batagov A Wu A Lai WB Sun Y Bee YM. A digital twin model incorporating generalized metabolic fluxes to identify and predict chronic kidney disease in type 2 diabetes mellitus. NPJ Digital Med. (2024) 7:140. doi: 10.1038/s41746-024-01108-6, PMID: 38789510 PMC11126707

[40] Sinha SK Shaheen M Rajavashisth TB Pan D Norris KC Nicholas SB. Association of race/ethnicity, inflammation, and albuminuria in patients with diabetes and early chronic kidney disease. Diabetes Care. (2014) 37:1060–8. doi: 10.2337/dc13-0013, PMID: 24550221 PMC4069363

[41] Mehrotra R Kermah D Fried L Adler S Norris K. Racial differences in mortality among those with CKD. J Am Soc Nephrol. (2008) 19:1403–10. doi: 10.1681/ASN.2007070747, PMID: 18385428 PMC2440295

[42] Lew QL Allen JC Nguyen F Tan NC Jafar TH. Factors associated with chronic kidney disease and their clinical utility in primary care clinics in a multi-ethnic southeast Asian population. Nephron. (2018) 138:202–13. doi: 10.1159/000485110 29253844

[43] Liu JJ Lim SC Yeoh LY Su C Tai BC Low S. Ethnic disparities in risk of cardiovascular disease, end-stage renal disease and all-cause mortality: a prospective study among Asian people with type 2 diabetes. Diabet Med. (2016) 33:332–9. doi: 10.1111/dme.13020, PMID: 26514089

[44] Lim CC He F Li J Tham YC Tan CS Cheng CY. Application of machine learning techniques to understand ethnic differences and risk factors for incident chronic kidney disease in Asians. BMJ Open Diabetes Res Care. (2021) 9:e002364. doi: 10.1136/bmjdrc-2021-002364, PMID: 34952839 PMC8710867

[45] Wang Y Chen X Song Y Caballero B Cheskin LJ. Association between obesity and kidney disease: a systematic review and meta-analysis. Kidney Int. (2008) 73:19–33. doi: 10.1038/sj.ki.5002586 17928825

[46] Nawaz S Chinnadurai R Al-Chalabi S Evans P Kalra PA Syed AA. Obesity and chronic kidney disease: a current review. Obes Sci Pract. (2023) 9:61–74. doi: 10.1002/osp4.629, PMID: 37034567 PMC10073820

[47] Zhang Y He D Zhang W Xing Y Guo Y Wang F. ACE inhibitor benefit to kidney and cardiovascular outcomes for patients with non-dialysis chronic kidney disease stages 3–5: a network meta-analysis of randomised clinical trials. Drugs. (2020) 80:797–811. doi: 10.1007/s40265-020-01290-3, PMID: 32333236 PMC7242277

[48] Hou FF Xie D Zhang X Chen PY Zhang WR Liang M. Renoprotection of optimal antiproteinuric doses (ROAD) study: a randomized controlled study of benazepril and losartan in chronic renal insufficiency. J Am Soc Nephrol. (2007) 18:1889–98. doi: 10.1681/ASN.2006121372, PMID: 17494885

[49] Billany RE Thopte A Adenwalla SF March DS Burton JO Graham-Brown MP. Associations of health literacy with self-management behaviours and health outcomes in chronic kidney disease: a systematic review. J Nephrol. (2023) 36:1267–81. doi: 10.1007/s40620-022-01537-0, PMID: 36645651 PMC10333418

[50] Adams RJ. Improving health outcomes with better patient understanding and education. Risk Manag Healthc Policy. (2010) 2010:61–72. doi: 10.2147/RMHP.S7500 PMC3270921 22312219

